# Risk Factors of Pneumonia Associated with Mechanical Ventilation

**DOI:** 10.3390/ijerph17020656

**Published:** 2020-01-19

**Authors:** Maria Kózka, Aurelia Sega, Katarzyna Wojnar-Gruszka, Agnieszka Tarnawska, Agnieszka Gniadek

**Affiliations:** 1 Department of Clinical Nursing, Faculty of Health Sciences, Jagiellonian University Collegium Medicum in Krakow, 31-501 Kraków, Poland; makozka@cm-uj.krakow.pl (M.K.); k.wojnar-gruszka@uj.edu.pl (K.W.-G.); 2 University Hospital in Krakow, 31-001 Kraków, Poland; agnieszkatarnawska@gmail.com; 3 Department of Epidemiological Nursing, Faculty of Health Sciences, Jagiellonian University Collegium Medicum in Krakow, 31-501 Kraków, Poland; agnieszka.gniadek@uj.edu.pl

**Keywords:** VAP, risk factors, intensive care unit, coexisting diseases

## Abstract

*Background:* The hospitalization of patients treated in the intensive care unit (ICU) in 5–15% of cases is associated with the occurrence of a complication in the form of ventilator-associated pneumonia (VAP). *Purpose:* Retrospective assessment of risk factors of VAP in patients treated at ICUs in the University Hospital in Krakow. *Methods:* The research involved the medical documentation of 1872 patients treated at the ICU of the University Hospital in Krakow between 2014 and 2017. The patients were mechanically ventilated for at least 48 h. The obtained data were presented by qualitative and quantitative analysis (%). The qualitative variables were compared using the *Chi*^2^ test. Statistically significant was the *p* < 0.05 value. *Results:* VAP was demonstrated in 23% of all patients treated in ICU during the analyzed period, and this infection occurred in 13% of men and 10% of women. Pneumonia associated with ventilation was found primarily in patients staying in the ward for over 15 days and subjected to intratracheal intubation (17%). A statistically significant was found between VAP and co-morbidities, e.g., chronic obstructive pulmonary disease, diabetes, alcoholism, obesity, the occurrence of VAP and multi-organ trauma, hemorrhage/hemorrhagic shock, and fractures as the reasons for admitting ICU patients. *Conclusions:* Patients with comorbidities such as chronic obstructive pulmonary disease, obesity, diabetes, and alcoholism are a high-risk group for VAP. Particular attention should be paid to patients admitted to the ICU with multi-organ trauma, fractures, and hemorrhage/hemorrhagic shock as patients predisposed to VAP. There is a need for further research into risk factors for non-modifiable VAP such as comorbidities and reasons for ICU admission in order to allow closer monitoring of these patients for VAP.

## 1. Introduction

Patients treated at intensive care units (ICU) have an increased risk of catching infections. The most common of them is ventilator-associated pneumonia (VAP). According to the new definition announced by the Center of Disease Control and Prevention (CDC), possible and probable VAP is understood as the deterioration of the mechanical ventilation parameters after about two days of stable ventilation, resulting in the increase of positive end expiratory pressure (PEEP) by at least 3 mm H_2_O and an increase in FiO_2_ by at least 20%. Patients have a temperature below 36 °C or above 38 °C and leucocytosis (≤12,000) or leukopenia (≥4000). The definition characterizes a possible VAP if there is a purulent secretion in the bronchial tree and a probable VAP if there is an increase in pathogenic pathogens in microbiological tests [1].

According to data from 2014, VAP occurs in about 5–15% of patients treated in ICUs [2] and mortality may be 70% [1]. In the European Centre for Disease Prevention and Control (ECDC) report, which included data from 2007 [3], the average incidence was lower than 7%, but the differences between European countries were large and ranged from 3% to 36%. In a subsequent ECDC report in 2014, the average incidence was 6% [4]. VAP risk factors may be related to the patient’s clinical condition, therapy, and staff activities [5]. The occurrence of complications is determined by many modifiable factors (e.g., body position, sedation, intubation and mechanical ventilation, upper airway instrumentation), as well as non-modifiable factors (e.g., age, duration of stay at the ward, comorbidities) [5,6].

Research indicates that the presence of foreign bodies like the endotracheal or gastric tubes are the main cause of VAP because they are most easily colonized by pathogenic agents [7,8,9]. It is very important to learn about other factors—very often unmodified—which predispose the patient to the occurrence of VAP (e.g., comorbidities). This knowledge will allow stricter observation of a patient who is more predisposed to VAP and enables early diagnosis in the case of emerging VAP symptoms.

Undoubtedly, the occurrence of VAP in a patient treated in ICUs significantly worsens the prognosis, increases the costs of treatment, and prolongs the time of hospitalization [5,10,11]. The purpose of the study is a retrospective assessment of risk factors of VAP in patients hospitalized in intensive care units.

## 2. Materials and Methods

The study covered 1872 patients hospitalized at the ICU of the University Hospital in Krakow between July 2014 to February 2017. In the analyzed period, there were 2059 patients in the ward. The inclusion criterion included hospitalization in ICUs for a minimum of two full days, therefore, 187 patients who were in the ward for a shorter time were not included in the study. The assessment was carried out on the third, eighth and fifteenth days of hospitalization.

The study used the analysis of medical records, which were the patients’ disease histories and TISS (therapeutic intervention scoring system) observation cards. The data was collected using a personal questionnaire.

The analysis criterion included gender, age, diagnosis, and co-morbidities. The analysis also included data on airway instrumentation in patients, the type and frequency of evacuated secretion, the presence of the oropharyngeal tube and the gastric tube, information on the length of intubation, duration of mechanical ventilation, and VAP diagnosis.

VAP was diagnosed following the definitions created by the CDC as well as the ECDC, taking into account microbiological diagnoses [12,13,14]. Permission to conduct the study was obtained from the Director of the University Hospital in Krakow, and all analyses of medical documentation were performed according to ethical standards and the principles of the Declaration of Helsinki.

The results were presented by qualitative and quantitative analysis (%). The qualitative variables were compared using the *Chi*^2^ test. The *p* < 0.05 value was assumed to be statistically significant.

## 3. Results

**Figure 1:**
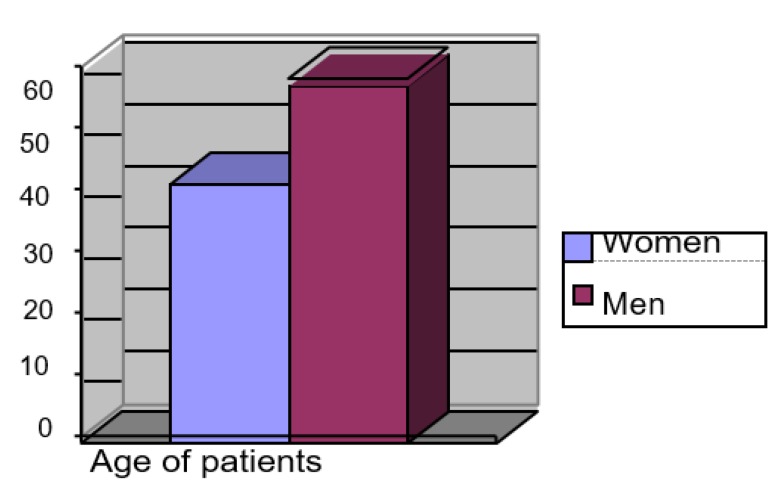
Percentage gender distribution of respondents.

**Figure 2:**
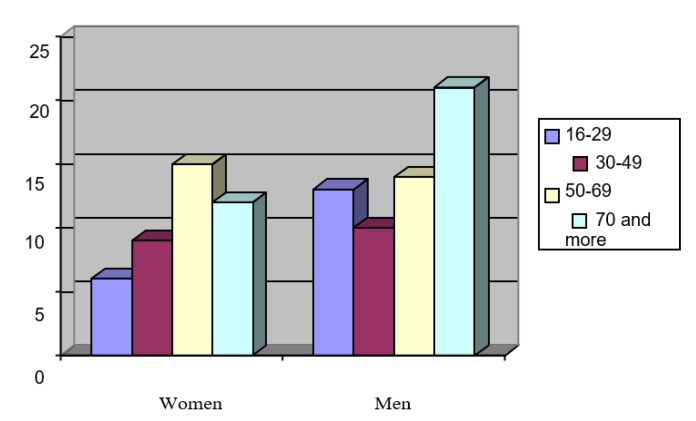
Percentage distribution of age of the respondents.

In the analyzed group, men prevailed *n* = 1094 (58%) in relation to women *n* = 778 (42%). The length of stay (LOS) in ICUs varied between 3 to 69 days. The average LOS of the respondents at the ICU was 3346 man-days. The average duration of the women’s stay was shorter than that of the men’s. The most common co-morbidities occurring among the studied patients were arterial hypertension *n* = 1216 (65%), diabetes *n* = 709 (38%), obesity *n* = 290 (15%), atherosclerosis *n* = 211 (11%), alcoholism *n* = 201 (11%), thromboembolism *n* = 161 (9%), and COPD *n* = 74 (4%). The characteristics of the studied group are presented in Figure 1 and Figure 2.

Data analysis showed a statistical significance between the length of hospitalization and the occurrence of VAP. The most common diagnosis was in patients staying in the ward for over 15 days (17%). In the group of patients treated within 3–7 days, only 1% were diagnosed with VAP.

In the group of analyzed comorbidities, highly statistically significant associations were found between the occurrence of such diseases as obesity, alcoholism, diabetes, and COPD and VAP (Table 1).

Also, the reasons for admission to the ICU have proven to be important in light of the possibility of VAP. The analysis showed a statistical significance between the occurrence of VAP and multi-organ injury (*p* = 0.000), fractures (*p* = 0.000), and hemorrhagic/hemorrhagic shock (*p* = 0.000), as direct causes of admitting patients to the ICU (Table 2).

Ventilator-associated pneumonia occurred in the majority of patients hospitalized due to the above-mentioned factors.

There was no statistically significant correlation between the presence of the oropharyngeal tube in intubated patients and the occurrence of VAP. The complication related to 1% of subjects (Table 3).

An extremely important issue was the type of airway instrumentation, and its relationship with the occurrence of VAP turned out to be statistically significant (*p* = 0.047; Table 4).

## 4. Discussion

Based on the results of our personal research, it has been shown that many factors influence the occurrence of pneumonia associated with mechanical ventilation in ICUs. These are mainly due to mechanical ventilation, airway instrumentation, and length of hospitalization. Similar results were provided by Bobik and Siemiątkowski [15], who showed that VAP occurs in 8–28% of mechanically ventilated patients. Furthermore, in patients treated at the ICU, this percentage increases to 27–30%, increasing the risk of complications by 3 to 10 times. The mentioned authors also showed that the use of mechanical ventilation and intubation causes a 21-fold increase in the incidence of VAP, and the risk increases steadily with each day of mechanical ventilation.

In our own study, it was shown that the length of hospitalization and the time of using mechanical ventilation had a significant influence on the occurrence of VAP. Similar results were presented by Karpel [16], which shows that the increased risk of VAP is dependent on the length of mechanical ventilation, and Szeter [17], indicating an increased risk of VAP by 1–3% for each day of mechanical ventilation. Also, the results of the research presented by Kubisz [18] are similar to the results of their own reports. The author emphasizes that when ICU hospitalization is prolonged beyond 72 h, the risk of pneumonia increases 12-fold.

In Rosenthal et al. [19], a report from 50 countries on VAP incidences ranged from 0.9 and 13.1 per 1000 ventilator-days. Ventilator-associated pneumonia density in the American CDC NHSN program from 2012 was, on average, 0.9 per 1000 ventilator-days [20]. In European countries, in a study published in 2012, the mean density of VAP was 8 in 1000 with intubation [1]. In another earlier study of Rosenthal et al., a report from 55 ICUs from 46 hospitals showed VAP incidences ranged from 10.0 to 52.7 per 1000 ventilator-days (the countries of southern America, Turkey, India) [21].

Noteworthy are the results regarding the relationship between co-morbidities and the occurrence of VAP. In the VAP study, this was found more frequently in patients with co-existing COPD, alcoholism, diabetes, and obesity. In the study by Rajnan et al. [22], VAP occurred in 57% of patients with diagnosed COPD. The results presented by Blot showed a relationship between the occurrence of VAP and diabetes [23].

In our study, there was a correlation between the reason for patient ICU admission and the occurrence of VAP. Patients with multi-organ trauma, hemorrhage/hemorrhagic shock and fractures more often suffered from VAP. The results of studies by Rajnan N. et al. [22] have also shown that the risk of developing VAP in patients with trauma was 76%.

There is a relationship between the type of airway instrumentation and the incidence of VAP. Patients with an inserted endotracheal tube are more likely to develop VAP than patients with a tracheotomy. In our research, pneumonia was more common in intubated patients (6%) than those with a tracheotomy (less than 0.5%). On the fifteenth day of hospitalization, VAP was diagnosed in the majority of intubated patients. Similar results were obtained in the research studies by Karaoglan et al. [24] in which the risk of VAP increased 7-fold among VAP patients, and 3-fold among patients with tracheotomies. Pirożyński et al. [25] showed similar relationships in their studies. He stressed that the patient’s intubation is one of the highest risk factors for VAP. According to the authors, the chances of the occurrence of pneumonia in the case of intubated patients increases by as much as 6 to 21 times.

In our study, there was no correlation between the presence of the oropharyngeal tube and VAP risk. In 1% of the patients intubated and in the gyroconeal section, VAP was diagnosed, while 24% were hospitalized with a laryngeal tube but did not have VAP.

The lack of association between the occurrence of pneumonia in patients treated in the ICU and the presence of laryngeal tubes may be associated with nurses administering oral cleaning in accordance with established procedures [23,24,25].

## 5. Conclusions

There is a need for further research into the risk factors of non-modifiable VAP, such as comorbidities, and the reasons for admission to ICUs in order to allow closer monitoring of these patients for VAP.

1. Patients with acute respiratory failure, multi-organ trauma, fractures, or hemorrhage/hemorrhagic shock are groups with a predisposition to the occurrence of VAP.
2. Particular attention should be paid to patients with comorbid COPD, obesity, diabetes, or alcoholism as high-risk groups for VAP.

## Figures and Tables

**Table 1:** Occurrence of VAP in selected comorbidities.

Comorbidities		VAP				
		Yes		No		*p*
		*n*	%	*n*	%	
**Diabetes**	Yes	161	9	548	29	0.016
	No	265	14	1181	48	
**Obesity**	Yes	145	8	145	8	<0.001
	No	281	15	1301	69	
**Alcoholism**	Yes	68	4	133	7	<0.001
	No	358	19	1313	70	
**COPD**	Yes	72	4	2	>0.5	<0.001
	No	354	23	1444	76.5	

COPD—chronic obstructive pulmonary disease, *p*—significance level, *n*—number of patients, %—percent.

**Table 2:** The occurrence of VAP vs. the cause of entering the ICU.

Reason for Stay at the ICU		VAP				
		Yes		No		*p*
		*n*	%	*n*	%	
**Multiple organ injury**	Yes	81	4	52	3	<0.001
	No	345	19	1394	74	
**Fractures, multiple fractures**	Yes	45	2	20	1	<0.001
	No	381	21	1426	76	
**Hemorrhage, hemorrhagic shock**	Yes	10	1	137	7	<0.001
	No	416	22	1309	70	

ICU—intensive care unit, *p*—significance level, *n*—number of patients, %—percent.

**Table 3:** The occurrence of VAP vs. the presence of an oropharyngeal tube in intubated patients.

Presence of Oropharyngeal Tubes	VAP Occurrence				Total		*p*
	Yes		No				
	*n*	%	*n*	%	*n*	%	
**Yes**	26	1	868	24	894	25	0.003
**No**	360	9	2434	66	2794	75	
**Total**	386	10	3302	90	3688	100	

VAP—pneumonia associated with mechanical ventilation, *p*—significance level, *n*—number of patients, %—percent.

**Table 4:** Occurrence of VAP vs. the type of airway breathing assist apparatus.

Type of Airway Breathing Assist Instrument	Occurrence of VAP				Total		*p*
	Yes		No				
	*n*	%	*n*	%	*n*	%	
**Intubation**	95	6	1315	88	1410	94	0.047
**Tracheotomy**	1	>0.5	83	6	84	6	
**Total**	96	6	1398	94	1494	100	

VAP—pneumonia associated with mechanical ventilation, *p*—significance level, *n*—number of patients, %—percent.

## Abbreviations

CDC	Centers for Disease Control
ECDC	European Centre for Disease Prevention and Control
ETT	Endotracheal Tube
HAP	Hospital-Acquired Pneumonia
ICU	Intensive Care Unit
VAP	Ventilator-Associated Pneumonia
LOS	length of stay
